# 

**Published:** 1887-02-26

**Authors:** 


The Hospital.

An Institution, Family, and Parochial Journal of Hospitals, Asylums, and all Agencies for the Care of the Sick, Criticism and News.

SATURDAY, FEBRUARY 26TH, 1887.

### The Literary Prize.

A PRIZE of **Two Guineas** will be given every month for the best story or incident based upon hospital, or asylum, or student life, or any incident connected with the professions of medicine or nursing.

### Hospital Housekeeping.

FORMS and full particulars of the Quilter Prizes under this head (see page 355 for 19th inst.) may be had on application to the Editor of THE HOSPITAL, Norfolk House, W.C. All competitive essays to be sent in by 31st March, 1887. Prizes, £10 10s. and £5 5s.

### THE CHILDREN'S CORNER.

### OUR PASTIMES.

**Children's Quarterly Prize Competition.**

Began 1st Jan., 1887: ends 26th Mar., 1887.

PRIZES.

FOR MOST CORRECT ANSWERS.

| | | | |
|---|---|---|---|
| 1st .. | Thirty Shillings | 3rd .. | Ten Shillings |
| 2nd .. | Twenty " | 4th .. | Five " |

FOR NEATEST ANSWERS.
Five Shillings.

FOR BEST LITTLE LETTERS.
Ten Shillings.

---

**Ninth Competition.**

No. 47. Conundrum. What is the difference between your Puzzle Editor and one of the Unemployed?

```
      *
    . * .
  . . * . .
* * * * * * *
  . . * . .
    . * .
      *
```

No. 48. Double Mesostich Puzzle. A letter near the middle of the alphabet; a domestic animal; dried fruit; a household commodity used to obtain a fire; a substance that produces unconsciousness; a product from China; the end of a kiss.

No. 49. An Arithmetical Question. What is the difference between half-a-dozen dozen, and six dozen dozen?

No. 50. Decapitation. Behead a relative pronoun and leave an article of headgear; behead an igneous element and leave anger; behead a fabric made of wool and leave unwilling.

No. 51. BURIED PROVERB. In each of the following lines is a word of a well-known proverb: Make up your mind. The horse eats hay. While I live I hope. I see the man. The chemistry of the sun. The metal shines.

**Results of Seventh Competition.**

No. 37. FIGURE-WORDS. Two thousand one hundred (MMC) *each* divided by (I), give us MIMIC.

No. 38. NUMERICAL ENIGMA. The answer here is February.

No. 39. CONUNDRUM. When one nigger dies, what do the others do? Why, of course they go a-black-berrying (a black burying).

No. 40. SQUARE WORD. The *lights* here are

S H I P
H I R E
I R O N
P E N S

No. 41. GEOGRAPHICAL PUZZLE. The river is the Thames.

No. 42. CONUNDRUM. This is a very old riddle. He was looking at *his own* portrait!

*All right*—P. S.; *Five right*—Alsie, Tim, Florrie, Mikado, Scrooge, A. C. K.; *Four right*—Peeler, Prospect, Skye, Joe, Dot, Ellie; *Three right*—Isobel, Paul Methven, Little Mary, Ariadne, Inez, Bismarck.

**Letters from Little Folk.**

The subject for next week's letter will be "Friends." Tell me whether your little companions are many or few, their tastes, and anything else you like.

Skye writes me the following interesting letter about her birthday. If I am not too late, may I add my congratulations to those of her many friends?

DEAR MR. EDITOR,—Yesterday was my birthday, and I had a very nice party with some of my school companions. Papa showed us his magic lantern. It lasted for about an hour. After tea we had a lot of games; about the best of them was a game called "Hissing and clapping." I got some very nice presents sent me from Edinburgh, and several from the visitors, and particularly a book, called *St. Clair of the Isles*, which seven of my school companions subscribed together for, and presented quite unexpectedly to me early in the morning. I think it was very kind of them. After supper, everyone went home about ten o'clock.—I remain, yours faithfully,

SKYE.

4, Milton Terrace, New Road, Chatham.

Here is Scrooge's story of a concert organised in aid of a poor old woman:—

DEAR MR. EDITOR,—I went to the concert on Monday, which I mentioned before. One of these concerts is given every year, in order to raise money for a poor old woman in the parish. She has worked very hard both in the church and school for over fifty years She is now old and infirm, and has no one to work for her, so she is quite dependent on these concerts, which they try to make as successful as they can. It was given in the schoolroom of the church, and was very full, so I hope they will get as much as last year, which was twenty-three pounds. Is it not a capital idea? I have no more time now, as I have all my lessons to do for to-morrow.

Yours truly, SCROOGE.

7, Charles Street, Pendleton, Manchester.

Florrie writes me the following interesting letter about a remarkably clever bow-wow:—

DEAR MR. EDITOR.—A short time ago, I went to a bazaar, held at a friend's house, near our own. They had a number of dogs, amongst which there was one named "Heren Happuck." In order to witness the tricks which this dog was then performing, I had to pay the large sum of two pence; then I entered the show-room, where the dog was about to begin its tricks, which were as follows:—No. 1. The dog was made to sit down, and then a piece of sugar was placed upon his nose; he remained in this posture for upwards of two minutes, and then, at a given signal from his mistress, tossed the sugar up in the air, caught it, and after that I saw it no more. No. 2 is perhaps the most extraordinary of all the tricks he did. His mistress pronounced these words, "Dead dog," over him, and he immediately lay stretched out on the ground, to all appearances as dead as a door-nail. Even when a piece of sugar was put close to his nose, he did not move a limb until he was told he might by his mistress. I have only related to you two of his many tricks, for if I were to relate them all my letter would be much too long: in fact, I think it is so now. I am very much pleased with the puzzles this week, and have done them all.

I am, yours very sincerely, FLORRIE.

20, St. John's Villas, East Dulwich Green.

**Letter Prize.**

One mark each: Skye, Isobel, P. S., Mikado, Tim, Peeler, Scrooge, Florrie, Ellie, Inez, Ariadne, and Little Mary.

**Notices to Correspondents.**

ELLIE.—Thank you very much for the puzzle.

FLORRIE.—Will you please to write on only one side of the paper?

MIKADO.—'Tis curious that "Mikado" should go to see "Mikado" played! I am sure you must have liked it. Thank you for enclosures.

TIM.—I am sure you must have quite a happy family Your note greatly interested me.

ISOBEL.—That must have been a delightful dance. How I should have liked to look in! What a pretty flower you painted in the corner of the letter you sent me!

PEELER.—Your letter was highly amusing. The lesson was certainly practical, but I fear a little severe on the poor partridge. Perhaps, you know, he did not eat so quickly as his companion.

Answers must be addressed to the "PUZZLE EDITOR," THE HOSPITAL, Norfolk House, Norfolk Street, Strand, W.C., not later than Thursday next. The "Children's Corner" Coupon (see advertisement pages) must be enclosed.

The address and full Christian name and surname, and the age of the competitor, must be stated in all replies. A *nom de plume* may be added for the purposes of publication.

### AMUSEMENTS WITH PROFIT.

### PRIZE COMPETITIONS.

**Quarterly Prize Competition.**

Began 22nd January; Ends 16th April.

**A Prize of Three Guineas**

will be awarded to the Competitor who shall have sent in the largest number of correct or best answers. No one will be permitted to win the Three Guinea Prize twice in any one year, but we shall award annually an additional

**Prize of Five Guineas**

to the competitor who shall have sent in the largest number of correct or best answers during the twelve months.

**No. 11. Double Acrostic.**

Think of *coals* for my primals,
And of *infirm* for my finals.

I was long the only *port* on Kiu-siu's shore,
Whither ships of foreign climes their commerce bore.

A *garden* beautiful, but where, none know,
The scene of human happiness and woe.

Dog-like, a member of a *feral race*,
Whom Edgar killed but failed quite to efface.

In Naples' Bay a favour'd summer *isle*,
Where Roman emperors their time would while.

In Devon, a *town* for carpets fam'd—
I'll stop, for the town I've pretty well named.

A *land* the prize for most strange custom wins,
To Europe sent some equally strange *twins*.

A great lexicographer's favourite *drink*,
Pope rhymed me with "nay," wrongly, I think.

Two daughters an old *king* of Britain had,
He gave them his kingdom; they drove him mad.

An *isle*, whose girth was a broad marshy fen,
Held the "last of the Saxons" and his men.

NO. 12. NURSE.

Give, in prose or verse, a definition of "Nurse." Credit will be given for answers which evince originality of thought in their composition. No answer may exceed one hundred words.

**Answers.**

No. 7. DOUBLE ACROSTIC.

D urha M
O dess A
C ronstad T
T ibe R
O ntari O
R oue N

Correct answers have been received from A. M. E., Mr. J. High, H. J. C., Ellice, Paddy, Mrs. Bedingfeld, Peeler, Ostrich, Patient, Condorcet, Mater, Gip, Gretta, K. M., Spiro, Perseverance, Miss Larkins, Tabs.

NO. 8. BURIED COMPOSERS.

The following are the names of the composers:—Paganini, Auber, Bridge, Handel, Costa, Flotow, Bach, Verdi, Arne, Wagner, Spohr, Weber, Glück.

Correct answers have been received from Miss Larkins, K. M., Gretta, Mater, Condorcet, Patient, Ostrich, Peeler, Mrs. Bedingfeld.

**Notices to Correspondents.**

A. M. E.—You may do as you suggest.

FREDERICK C.—You will not find Orinoco, "mid the Canadian Lakes."

Answers must be addressed to THE PRIZE EDITOR, AMUSEMENTS WITH PROFIT, THE HOSPITAL, Norfolk House, Norfolk Street, Strand, W.C., not later than Saturday next. The full name and address must in all cases be given, but a *nom de plume* may be added for publication. Only one side of the paper should be written on. The "Amusements with Profit" Coupon (see advertisement pages) must be enclosed with replies.

### The In- and Out-Patients' Hospital Diary.

This Diary is so arranged as to make some portion of it appear every week, and the whole in each monthly part. It has been very difficult to compile, and corrections will at all times be gratefully received. It has been suggested that similar information about the larger Provincial and Scotch Hospitals would be useful to many readers. We should be glad to hear if this is felt to be the case.

### DISEASES AND IRREGULARITIES OF THE TEETH.—*continued.*

| Hospitals and Terms of Admission. | Physicians, Surgeons, and Medical Officers, with Days and Hours of Attendance. |
|---|---|
| New Hospital for Women, 222, Marylebone rd., W. | *Surgeon*, Mr. H. Apperley |
| North-West London Hosp., 18, Kentish Town Rd., N.W. | *Surgeon*, Mr. W. A. Maggs, F. 9 |
| Paddington Green Children's Hospital, Paddington | *Surgeon*, Mr. C. Vincent Cotterell, W. 9 |
| Royal Free Hospital, Gray's Inn Road, W.C. | *Surgeon*, Mr. H. Harris, M. Th. |
| Royal Hospital for Children and Women, Waterloo Bridge Road, S.E. | *Surgeon*, Mr. Whitehouse, F. 1.30 |
| St. Bartholomew's Hospital, Smithfield, E.C. | *Surgeons*, Messrs. Ewbank, Tu.; Paterson, F.; Ackery, F.; Mackrett, Tu. Hour, 9 |
| St. George's Hospital, Hyde Park Corner, S.W. | *Surgeon*, Mr. Winterbottom, Tu. S. 9 |
| St. Mary's Hospital, Cambridge Place, Paddington | *Susgeon*, Mr. H. Hayward, W. S. 9.30 |
| St. Thomas's Hospital, Westminster Bridge Road, S.E. | *Surgeon*, Mr. Truman, Tu. F. 10 |
| University College Hospital, Gower Street, W.C. | *Surgeon*, Mr. S. J. Hutchinson, W. 9.30 |
| Victoria Hospital for Children, Chelsea, S.W. | *Surgeon*, Mr. F. Fox, S. 9.0 |
| West London Hospital, Hammersmith Road, W. | *Surgeon*, Mr. H. L. Albert, Tu. F. 9.30 |
| Westminster Hospital, Broad Sanctuary, S.W. | *Surgeons*, Messrs. J. Walker and M. A. Smale, W. S. 9.15 |

### THROAT DISEASES.

| Hospitals and Terms of Admission. | Physicians, Surgeons, and Medical Officers, with Days and Hours of Attendance. |
|---|---|
| Central London Throat and Ear Hospital, Gray's Inn Road, W.C. | *Surgeons. See* "Ear Cases." M. W. Th. S. 2.30; Tu. F. 5 |
| Great Northern Central Hospital, Caledonian Rd, N. | *Surgeon*, Mr. Spencer Watson, W. 2 |
| Guy's Hospital, St. Thomas Street, S.E. | *Surgeon*, Mr. Symonds, Th. 1 |
| Hospital for Diseases of the Throat and Chest, 32, Golden Sq. Hon. Sec., Mr. G. C. Witherby. Poor free; others by small payment | *Physicians*, Drs. Wolfenden, Tu. F. 2.30; Macdonald, Tu. F. 6.30; Bond, W. S. 2.30<br>*Surgeon*, Mr. T. Mark Hovell, M. Th. 2.30 |
| King's College Hospital, Portugal Street, W.C. | *Surgeon*, Mr. Cartwright, Tu. F. 10 |
| Middlesex Hospital, Berners Street, W. | *Surgeon*, Mr. Hensman, Tu. 9 |
| North West London Hospital, 18, KentishTn. Rd., N.W | *Surgeon*, Mr. Collier, M. 1.30 |
| St. Bartholomew's Hospital, Smithfield, E.C. | *Surgeon*, Mr. Butlin, F. 2.30 |
| St. George's Hospital, Hyde Park Corner, S.W. | *Surgeon*, Dr. Whipham, Th. 1.30 |
| St. Mary's Hospital, Cambridge Place, Paddington | *Surgeon*, Mr. A. T. Norton, M. Th. 9.30 |
| St. Thomas's Hospital Westminster Bridge Road, S.E. | *Physician*, Dr. Semon, Tu. F. 1.30 |
| University College Hospital, Gower Street, W.C. | *Physician*, Dr. Poore, Th. 1.30 |
| Westminster Hospital, Broad Sanctuary, S.W. | W. 9. *See* General Hospitals. |

### DISEASES OF WOMEN, (OBSTETRIC CASES, ETC.)

| Hospitals and Terms of Admission. | Physicians, Surgeons, and Medical Officers, with Days and Hours of Attendance. |
|---|---|
| Charing Cross Hospital, West Strand, W.C. | *Physicians*, Drs. J. W. Black, Amand Routh, Tu. F. 1.30 |
| Chelsea Hospital for Women, Fulham Road, S.W. Sec., Mr. J. H. Easterbrook. Payment from 10*s.* 6*d.* a week. Poor by Governor's letter. Out-patients, if without subscriber's letter, pay 6*d.* each attendance | *In-Physicians*, Drs. J. H. Aveling, M. Th.; A. W. Edis, Tu. F.; F. Barnes, W. S. Hour, 2.30<br>*Out-Physicians* Drs. Dickinson and Mackeron, M.; W. Travers and G. Harper, Tu.; F. Jones and J. I. Parsons, W.; and H. T. Rutherford, F. Hour, 2 |

### DISEASES OF WOMEN, (OBSTETRIC CASES, ETC.)—*Continued.*

| Hospitals and Terms of Admission. | Physicians, Surgeons, and Medical Officers, with Days and Hours of Attendance. |
|---|---|
| East London Hospital for Children and Dispensary for Women, Shadwell, E. A Subscriber's letter is required, except in urgent cases | *Out-Physicians*, M. Tu. W. Th. F. 1 and 3; Tu. F. 9. *See* "Children's Diseases." |
| German Hospital, Dalston Lane, Dalston, E. | *Physician*, Dr. Rasch, M. Th. 9 |
| Great Northern Central Hospital, Caledonian Rd., N. | *Physician*, Dr. F. Barnes, W. 2 |
| Grosvenor Hospital for Women and Children, 3 and 4, Vincent Square, S.W. | *Out-Physicians and Surgeons*, Tu. W. Th. S. 2. *See* "Children's Diseases." |
| Guy's Hospital, St. Thomas Street, S.E. | *Physicians*, Drs. Galabin, Horrocks, M. Tu. F. 1.30 |
| Hospital of St. John and St. Elizabeth, 47, Gt. Ormond Street, W.C. For females suffering from long standing disease. | *Physicians*, Drs. Constable and Tegart (no out-patients) |
| Hospital for Women, 29 and 30, Soho Sq., W. Sec., Mr. David Cannon. In-patients admitted free by Governor's letter; also by payment. Out-patients free | *In-Physicians*, Drs. Carter, W. S. 2; R. T. Smith, Tu. F. 2; E. Holland, Tu. F. 9.30<br>*In-Surgeon*, Mr. H. A. Reeves, M. 4, Th. 2;<br>*Out-Physicians*, Drs. R. T. Smith, F. 10; E. Holland, W. 10; Mansell-Moullin, M. Th. 11; Bedford Fenwick, Tu. 10; J. Oliver, S. 10<br>*Out-Surgeon*, Mr. S. Osborn, Th. 2 |
| King's College Hospital, Portugal Street, W.C. | *Physician*, Dr. Playfair, Tu. Th. S. 2 |
| London Hospital, Whitechapel Road, E. | *In-Physician*, Dr. Herman, Tu. F. 1.30<br>*Out-Physician*, Dr. Lewers, W. S. 1.30 |
| Metropolitan Free Hospital, Gray's Inn Road, W.C. | *Physician*, Dr. A. Venn, W. 2 |
| Middlesex Hospital, Berners Street, W. | *In-Physician*, Dr. Edis, Tu. F. 1.30<br>*Out-Physician*, Dr. Duncan, W. S. 1.30 |
| New Hospital for Women, 222, Marylebone Road, W. Hon. Sec., Miss C. Vincent. By subscriber's letter. Out-patients pay 6*d.* entrance fee, and 2*d.* each visit. | *In-Physicians*, Drs. Anderson, Atkins, Marshall, de la Cherois<br>*Out-Physicians*, Drs. Marshall, De la Cherois, Hitchcock. Daily, 1 to 3, and S 9 to 10. (All the physicians are women) |
| North-West London Hospital, 18, KentishTn. Rd., N.W | W. 1.30. *See* General Hospitals |
| Royal Free Hospital, Gray's Inn Road, W.C. | *In- and Out-Physician*, Dr. Hayes, Tu. S. 9 |
| Royal Hospital for Children and Women, Waterloo Bridge Road, S.E. Sec., Mr. R. G. Kestin. | *Out-Physicians*, Daily at 2<br>*Out-Surgeon*, W. 2<br>*See* "Children's Diseases." |
| St. Bartholomew's Hospital, Smithfield, E.C. | *In-Physician*, Dr. M. Duncan, Tu. Th. 2<br>*Out-Physician*, Dr. Godson. W. S. 9 |
| St. George's Hospital, Hyde Park Corner, S.W. | *Physician*, Dr. Champneys, Th. 1.30 |
| St. Mary's Hospital, Cambridge Place, Paddington, W. | *Physicians*, Drs. Meadows, Tu. F. 9.30; H. Jones, M. Th. 1.30 |
| St. Thomas's Hospital, Westminster Bridge Road, S.E. | *Physician*, Dr. Jervis, Tu. F. 2 |
| Samaritan Free Hospital for Women and Children, Lower Seymour Street, W. Branch, 1, Dorset Street, Manchester Square, W. Sec., Mr. Geo. Scudamore. Governor's letter or card is required if the disease of the applicant is *not* peculiar to her sex. Out-patients department (free) is at Edward's Mews, Duke Street, W. | *In-Physicians*, Drs. P. Boulton, M. Prickett, Amand Routh. Daily, 2<br>*In-Surgeons*, Messrs. G. G. Bantock, J. H. Thornton, W. A. Meredith. Daily, 9.30<br>*Out-Physicians*, Drs. M. Prickett, and Amand Routh, W. S. 1.30<br>*Out-Surgeons*, Messrs. W. A. Meredith, and J. D. Malcolm, M. Th.; A. H. G. Doran, and W. S. A. Griffith, Tu. F. Hour 1.30 |
| University College Hospital, Gower Street, W.C. | *Physicians*, Drs. G. Hewitt, M. Th. 1.30; J. Williams, Tu. F. 2 |
| West London Hospital, Hammersmith Road, W. | *Physicians*, Drs. A. Venn, Moullin, W. S. 2 |
| Westminster Hospital, Broad Sanctuary, S.W. | *Physicians*, Drs. Potter, Tu. F. 2; Grigg, Tu. F. 9 |

